# Evaluating the Prognostic Value of Kansas City Cardiomyopathy Questionnaire (KCCQ) Scores for 6‐Month Readmissions in Southeast Asian Populations With Heart Failure

**DOI:** 10.1002/clc.70136

**Published:** 2025-07-02

**Authors:** Jeanne SY Ong, Ming Fatt Kham, Jonah Goh, Francis Phng, Po Fun Chan, Poay Huan Loh, Christine Wu

**Affiliations:** ^1^ Department of Medicine Ng Teng Fong General Hospital Singapore Singapore; ^2^ Health Services Research & Analytics Ng Teng Fong General Hospital Singapore Singapore

**Keywords:** congestive heart failure, data modeling, machine learning, patient reported outcome measures, testing and evaluation

## Abstract

**Background:**

Heart failure (HF) is a prevalent cause of hospital readmissions. Our study aims to determine the correlation between the Kansas City Cardiomyopathy Questionnaire (KCCQ) scores and 6‐month readmission in our Southeast Asian population.

**Methods:**

We evaluated KCCQ‐12 in a cohort of 180 patients at first post‐discharge visit after a recent hospitalization for HF with reduced ejection fraction (HFrEF). Logistic regression was used to determine the predictive significance of the KCCQ scores for 6‐month HF readmission. The selection of predictive parameters was performed using Stepwise Akaike Information Criterion (StepAIC).

**Results:**

Out of 180 patients, 52 (29%) were readmitted for HF within 6 months. The mean KCCQ score was higher in the non‐readmitted group (78.5) compared to the readmitted group (69.7, *p* = 0.0129). Multivariate analysis indicated a significant association between higher KCCQ scores (better health status) and lower HF readmission rates (adjusted OR = 0.929, *p* = 0.0255). The initial predictive model, using patient demographic data, had an AUC score of 0.64. Integrating KCCQ scores with demographics, length of stay (LOS), medical history and discharge medication variables raised the AUC score to 0.82.

**Conclusion:**

KCCQ scores recorded at first post‐discharge encounter were found to have a significant relationship with 6‐month readmissions in our cohort, suggesting that KCCQ scores can serve as an effective clinical indicator of 6 month readmissions.

## Graphical abstract

Stepwise model selection using the Akaike Information Criterion was employed to create a streamlined predictive model that included only the most significant variables. The predictive power of the KCCQ overall score was assessed within this optimized model. The model performance suggests that KCCQ‐12 is effective in predicting 6‐month HF readmissions.

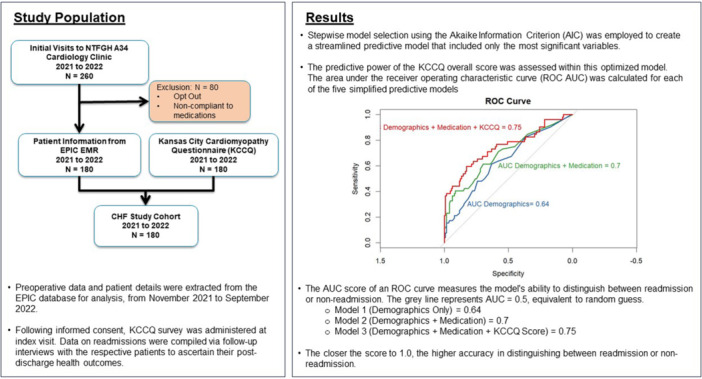

## 1 Introduction

Heart Failure (HF) is a global epidemic affecting more than 64 million people worldwide [1]. Singapore has a significant proportion of population either at risk or suffering from HF [2], with a prevalence considerably higher (4.5%) than in the US and Europe (1%−2%) [2, 3]. Globally, it is one of the leading causes of hospital admission particularly in individuals over the age of 65 [4]. In Singapore, it remains a top cause of mortality accounting for approximately 21.8% of all deaths from 2020 to 2022 [5]. The rising burden of HF can be jointly attributed to an aging population and concomitant increase in prevalence of risk factors such as hypertension, coronary artery disease, diabetes, and obesity [4, 5, 6, 7]. Whilst advancements in HF treatment and care have led to improved patient outcomes and survival, this has paradoxically resulted in greater strain on the healthcare system due to costs of long‐term management, therapeutics and readmissions [8, 9, 10]. It is therefore important to recognize and identify HF patients who have a higher likelihood of hospital readmissions and may benefit from closer follow up or further optimization by the medical.

Validated in various populations worldwide [11, 12], the Kansas City Cardiomyopathy Questionnaire (KCCQ) is a patient‐reported outcome measure (PROM) tool that essentially captures how HF affects patients' functional status and quality of life (QoL). It has also been utilized as a predictive tool for short term (30‐day) HF readmissions [11]. Furthermore, because the KCCQ was developed and largely validated in Western populations, its accuracy in Asian populations require further validation given the heterogeneity [13, 14].

With the aforementioned considerations, we evaluated the utility of KCCQ‐12, combined with clinical variables, in predicting 6‐month readmissions in Singapore's HF population [15], one that is multiracial and multicultural.

## 2 Methods

This prospective cohort study was conducted at Ng Teng Fong General Hospital Singapore between November 2021 to September 2022, and approved by the local hospital's Institutional Review board. Inclusion criteria were patients with recent admission for acute HF attending a first post‐discharge clinic encounter (index visit), aged 18 years old and above, left ventricular ejection fraction (LVEF) ≤ 40% on most recent echocardiogram, without significant chronic kidney disease and/or cognitive impairment. Exclusion criteria included patients who did not successfully complete the KCCQ‐12, non‐compliance to medications, withdrawal from the study, lost to follow up, inability to understand study procedure and inability to provide informed consent.

For every patient who met the inclusion criteria and consented to the study, a trained study coordinator would administer the KCCQ‐12 during the index visit. Follow‐up interviews were conducted to ascertain their post‐discharge health outcomes. The primary outcomes were readmission for HF within 6 months of index visit, and KCCQ‐12 scores. Anonymized HF patient data including demographics, clinical variables, medications and readmission details were extracted from the hospital database. Comorbidities were assessed using the Charlson Comorbidity Index (CCI).

### 2.1 Statistical Analysis

#### 2.1.1 Univariable and Multivariable Analyses

Initial univariate analysis compared outcomes between readmitted and non‐readmitted groups, such as demographics, comorbidities, discharge medications and KCCQ‐12 scores. The *t*‐test and Likelihood Ratio Tests (LRT) were used for continuous and for categorical variables respectively [16]. A multivariate logistic regression model analysis was then performed to investigate how patient demographics, comorbidities, medications and KCCQ‐12 scores were independently associated with 6 month readmission.

#### 2.1.2 Model Development

**Figure 1:**
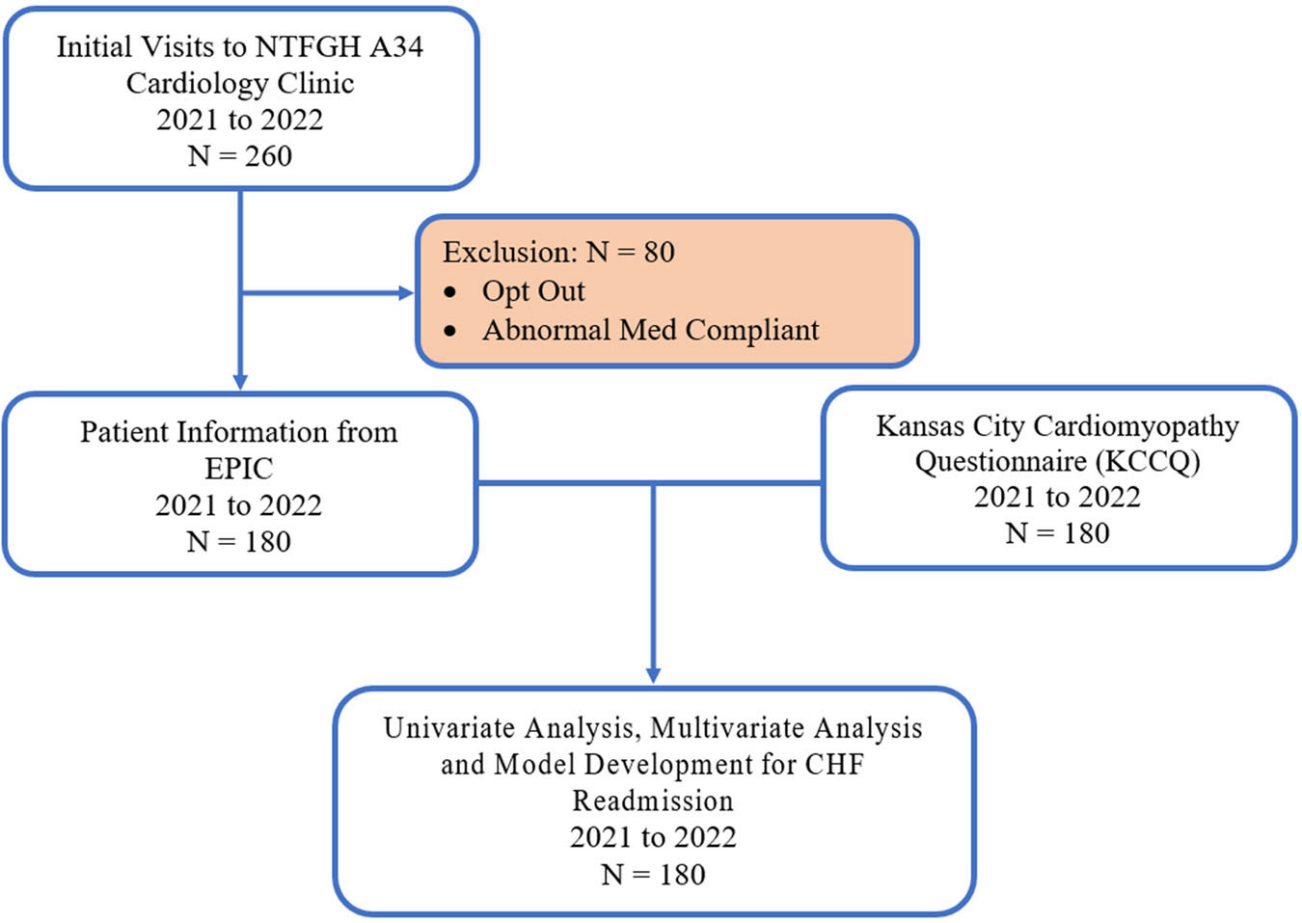
Identification of the study cohort. Process map of how the training data sets were prepared from the original data source for the development of the ML models.

Using the Akaike Information Criterion (AIC) [17], 4 simplified predictive models were created with stepwise model selection including clinical variables that were statistically significant. The data was then randomly divided into two sets: 20% in the test data set and 80% in the training data set. The test data set was used to fit and develop the prediction model, which was subsequently used for validation with the training data set. Figure 1 depicts the process of how the original data set was split into the Training and Test sets for model development. The predictive accuracy of each model was determined statistically by analyzing the area under the receiver operating characteristic curve (ROC) [18]. The four models was further divided into two submodels each, where one included KCCQ‐12 as a variable. The importance of KCCQ‐12 was then analyzed by comparing the ROCs between these two submodels, repeated for all four models. Modeling analysis was performed using StepAIC function from the MASS package in R.

## 3 Results

The study initially screened 260 patients however 80 patients were excluded for non‐compliance (Figure 1). Of the 180 HF patients enrolled in the study, 52 patients (28.9%) were readmitted within 6 months.

### 3.1 Univariate Analysis

#### 3.1.1 Demographic and Clinical Characteristics

Comparing the demographic profile between non‐readmitted and readmitted groups, there were no differences in mean age (61.0 vs. 62.3 years, *p* = 0.601) and racial profile distribution. However there was significantly more female patients in the readmitted group as compared to the non‐readmitted group (40.4 vs. 21.1%, *p* = 0.027). The length of hospital stay for the index admission was significantly longer in the readmitted group (6.06 vs. 4.14 days, *p* = 0.010), and there was a higher percentage of patients admitted in Class “C” subsidized wards (96.2 vs. 81.3%, *p* = 0.004) as compared to the non‐readmitted group. There were no significant differences in comorbidities as measured by the CCI between both groups (Table 1).

**Table 1:** Summary of demographics, medical history and outcomes between HF readmission and non‐readmission within 6 months.

Demographic characteristic	Readmission within 6 months after discharge		
	No (*n* = 128)	Yes (*n* = 52)	*p* value
Age, years, Mean (SD)	61.0 (14.5)	62.3 (15.9)	0.6010
LOS at encounter, days, Mean (SD)	4.14 (3.60)	6.06 (5.66)	0.0102
No. prior admission	0.41 (1.07)	1.06 (1.39)	0.0043
*Ward category*			0.0043
Class C (subsidized)	104 (81.3)	50 (96.2)	
Others	24 (18.8)	2 (3.8)	
*Race*			0.7836
Chinese	78 (60.9)	29 (55.8)	
Indian	11 (8.6)	4 (7.7)	
Malay	21 (16.4)	12 (23.1)	
Others	18 (14.1)	7 (13.5)	
*Gender*			0.0265
Male	101 (78.9)	31 (59.6)	
Female	27 (21.1)	21 (40.4)	
*Comorbidity*			
Myocardial Infarction	28 (21.9)	11 (21.2)	0.9150
Congestive Heart Failure	95 (74.2)	38 (73.1)	0.8744
Peripheral Vascular Disease	3 (2.3)	2 (3.8)	0.5820
Cerebrovascular Disease	6 (4.7)	1 (1.9)	0.4000
Chronic Pulmonary Disease	8 (6.3)	2 (3.8)	0.5270
Peptic Ulcer Disease	3 (2.3)	0 (0.0)	0.9860
Mild Liver Disease	7 (5.5)	1 (1.9)	0.3170
Diabetes without Chronic Complications	54 (42.2)	15 (28.8)	0.0974
Diabetes with Chronic Complications	70 (54.7)	28 (53.8)	0.9182
Hemiplegia or Paraplegia	3 (2.3)	1 (1.9)	0.8620
Renal Disease	36 (28.1)	15 (28.8)	0.9220
Malignancy without metastasis	1 (0.8)	0 (0.0)	0.9880
At least 1 CCI	111 (86.7)	49 (94.2)	0.1579
*Without the following comorbidity:*			
Coronary Artery Disease (CAD)	50 (39.0)	14 (26.9)	0.1253
Hypertension	95 (74.2)	39 (75.0)	0.9133
Stroke	0 (0.0)	1 (1.9)	0.9860
Obesity	15 (11.7)	4 (7.7)	0.4290
*PROMs*			
KCCQ Score, Mean (SD)	78.5 (19.7)	69.7 (22.7)	0.0129
*Discharged medication*			
Hydralazine	8 (6.3)	13 (25.0)	0.0009
Betablocker	128 (100.0)	50 (96.2)	0.9870
Bisoprolol	123 (96.1)	42 (80.8)	0.0022
Carvedilol	22 (17.2)	15 (28.8)	0.0824
Atenolol	5 (3.9)	2 (3.8)	0.9850
ACE/ARB/ARNI	118 (92.2)	46 (88.5)	0.4290
Entresto	70 (54.7)	25 (48.1)	0.4212
Valsartan	45 (35.1)	16 (30.8)	0.5730
Lisinopril	23 (18.0)	8 (15.4)	0.6780
Enalapril	20 (15.6)	10 (19.2)	0.5570
Losartan	15 (11.7)	11 (21.2)	0.1070
Irbesartan	3 (2.3)	1 (1.9)	0.8620
Telmisartan	1 (0.8)	2 (3.8)	0.1890
SGLT2‐I	101 (78.9)	36 (69.2)	0.1700
Empagliflozin	78 (60.9)	26 (50.0)	0.1795
Dapagliflozin	26 (20.3)	12 (23.1)	0.6810
MRA	98 (76.6)	37 (71.2)	0.4483
Spironolactone	98 (76.6)	37 (71.2)	0.4483
Nitrate	12 (9.4)	14 (26.9)	0.0035
Isosorbide mononitrate	7 (5.5)	5 (9.6)	0.3180
Isosorbide dinitrate	6 (4.7)	10 (19.2)	0.0040

*Note:* Numbers in the parenthesis are percentage except indicated. Medication prescription data was extracted from Epic EMR database records based on historical medication orders.

#### 3.1.2 KCCQ‐12 Scores

The mean KCCQ‐12 score was higher in the non‐readmitted group as compared to the readmitted group (78.5 vs. 69.7, *p* = 0.013) (Table 1).

#### 3.1.3 Discharge Medications

More patients in the non‐readmitted group were prescribed bisoprolol on discharge (96.1 vs. 80.8%, *p* = 0.002), whilst other medications including hydralazine (25.0 vs. 6.3%, *p* = 0.001), isosorbide dinitrate (19.2 vs. 4.7%, *p* = 0.004) and nitrate (26.9 vs. 9.4%, *p* = 0.004) were more frequently prescribed on discharge in the readmitted group.

### 3.2 Multivariate Analysis

Multivariate analysis demonstrated a few significant variables that were either positively or negatively associated with 6 month readmission (Table 2). Patients who were discharged with bisoprolol (OR 1.141, *p* = 0.043) and Carvedilol (OR 1.318, *p* = 0.001) had higher odds of 6 month readmission. On the other hand, a 1 unit increase in KCCQ‐12 score was significantly associated with a 2% reduction in odds of 6 month readmission (OR 0.980, *p* = 0.040). Other variables that were negatively associated with 6 month readmission included diabetic patients without complications (OR 0.285, *p* = 0.012), and the absence of coronary artery disease (OR 0.232, *p* = 0.006)

**Table 2:** Prognostic value of readmission within 6 months after discharge of combination of models.

Models	ROC area	ROC increase (%)
Group 1: Patient Demographics with KCCQ		
Age + Gender + Race	0.62	
Age + Gender + Race+ KCCQ	0.65	4.8%
Group 2: Patient Medical History with KCCQ		
Diabetes without Chronic Complications + NO.CAD	0.64	
Diabetes without Chronic Complications + NO.CAD + KCCQ	0.70	9.4%
Group 3: LOS and Medications with KCCQ		
Isosorbide dinitrate + Carvedilol + LOS Encounter	0.60	
Isosorbide dinitrate + Carvedilol + LOS Encounter + KCCQ	0.68	13.3%
Group 4: All Factors with KCCQ		
Patient Demographics + Patient Medical History + Diagnosis	0.79	
Patient Demographics + Patient Medical History + Diagnosis + KCCQ	0.82	3.8%

### 3.3 Predictive Model

To predict the risk of 6 month readmission, four main models were developed using different combinations of variables including KCCQ‐12 scores, medications (Bisoprolol, Isosorbide dinitrate, Carvedilol, Telmisartan), patient demographics (age, gender, race, Ward Category), length of stay, and comorbidities (CCI index) (Table 3).

**Table 3:** Summary of multivariate analysis investigating the effects of demographic characteristics and overall KCCQ score on readmission rate within 6 months.

Demographic characteristic	Odds ratio	Standard error	*p* value
KCCQ	0.980	0.010	0.040
Bisoprolol	1.141	0.065	0.043
Isosorbide dinitrate	1.691	0.191	0.006
Carvedilol	1.318	0.086	0.001
Telmisartan	2.116	0.639	0.241
Ward Category	0.332	0.803	0.170
LOS	1.066	0.020	0.002
Peripheral Vascular Disease	5.574	1.157	0.137
DM without Chronic Complications	0.285	0.498	0.012
No History of CAD	0.232	0.530	0.006
Normotensive	2.241	0.525	0.124
SGLT2‐I	1.117	0.074	0.133

**Figure 2:**
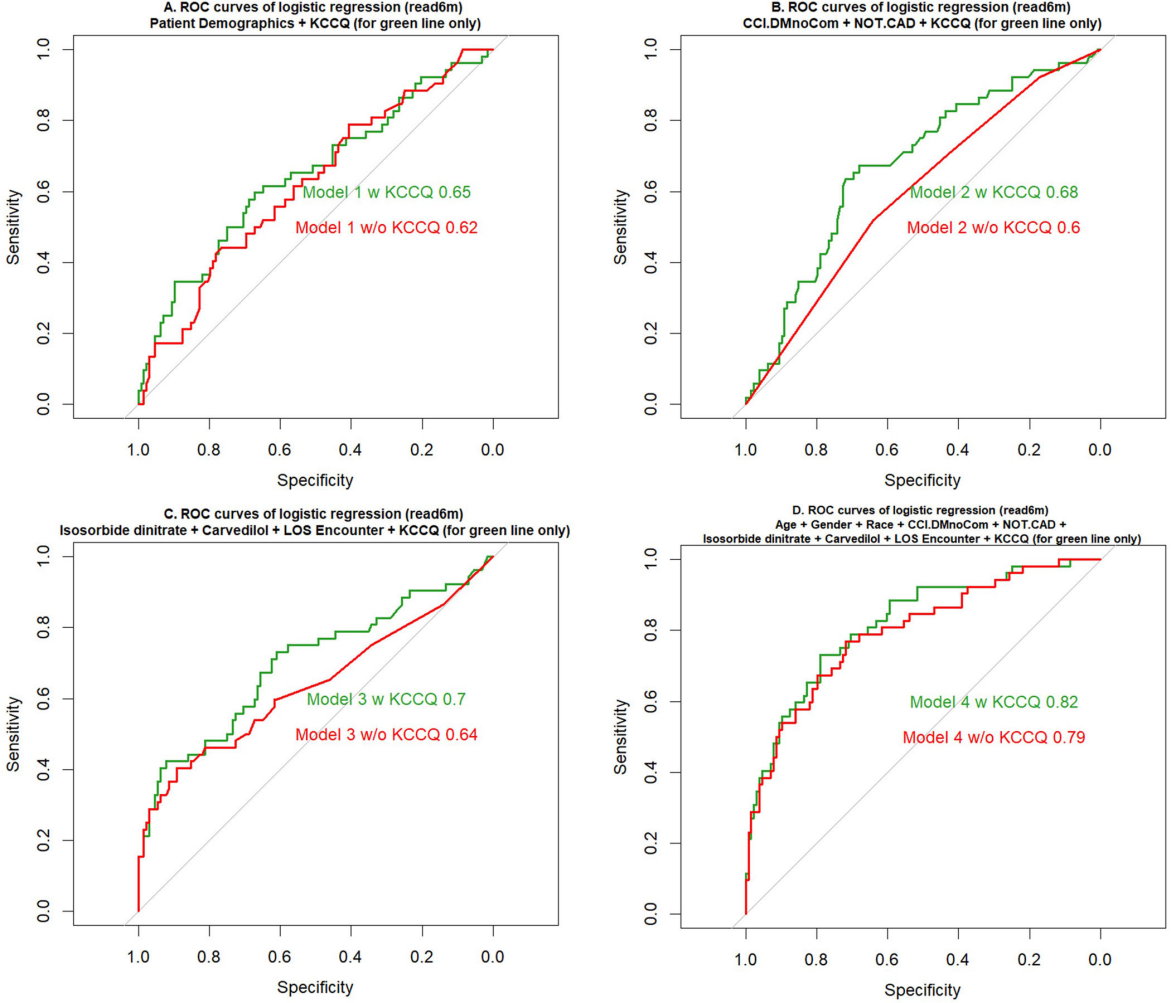
Comparison of receiver operating curve. comparison of ROC area. Green line is ROC curve of model with KCCQ and red line is ROC curve of model without KCCQ. (A) ROC curves of logistic regression (read6m) = Age + Gender + Race + KCCQ (for green line only). (B) ROC curves of logistic regression (read6m) = Diabetes without Chronic Complications + NO.CAD + KCCQ (for green line only). (C) ROC curves of logistic regression (read6m) = Isosorbide dinitrate + Carvedilol + LOS Encounter + KCCQ (for green line only). (D) ROC curves of logistic regression (read6m) = Age + Gender + Race + Diabetes without Chronic Complications + NO.CAD + Isosorbide dinitrate + Carvedilol + LOS Encounter + KCCQ (for green line only.

Amongst the four models, model 4 (patient demographics, comorbidities, medications, length of hospital stay) had the best predictive accuracy of 6 month readmission with a ROC of 0.79. The addition of KCCQ scores consistently increased the model accuracy across all 4 models, with ROC improvements of 4.8%, 9.4%, 13.3% and 3.8% for models 1, 2, 3, and 4 respectively. With this, model 4 remained as the best model after addition of KCCQ‐12 score with a ROC of 0.82 (Figure 2).

## 4 Discussion

### 4.1 Impact of KCCQ‐12

Our findings support existing data that KCCQ‐12 score is a robust tool in identifying patients at risk of HF readmissions [11, 12, 13]. Although no consensus exists in interpreting KCCQ scores, health status category is commonly assigned based on quartiles (0−24 = very poor to poor health status, 25−49 = poor to fair; 50−74 = fair to good; 75−100 = good to excellent). In our study, the mean score for readmitted patients (69.4) fell into the “fair to good” quartile while that of non‐readmitted patients (78.5) in the “good to excellent” quartile. Patients with scores 50 to 74 have been shown to have a RR of death or hospitalization of 1.5 as compared to those of scores above 75 [19]. However, in our study both groups had a wide range of KCCQ scores and was not powered to establish a clear cut‐off value predicting readmission.

### 4.2 Medications

Betablocker, ACE‐I/ARB/ARNI, SGLT2‐inihibitors and MRA were more commonly prescribed in the non‐readmitted group, although these differences were not statistically significant.

Betablockers were the most commonly prescribed medication, the majority receiving bisoprolol (*n* = 165). All of the 128 non‐readmitted patients were prescribed betablockers on discharge compared to 96.2% in the readmitted group; however this was not statistically significant. Non‐usage of betablockers has been shown to predict readmissions in Asian populations [20]. In our cohort a significant difference was only observed in the bisoprolol group. (96.2% vs. 80.0%, *p* = 0.0022).

Nitrates and hydralazine were more commonly prescribed in the readmitted group. These vasodilators are more commonly prescribed in patients who are unable to tolerate ACE/ARB/ARNI such as those with renal impairment or those with residual obstructive coronary artery disease, both of which portend poorer outcomes. The absolute numbers in our population however, are small (ISDN *N* = 16; Hydralazine *N* = 21).

### 4.3 Co‐Morbidities

The ASIAN‐HF study [20] found a history of chronic kidney disease, diabetes mellitus and coronary artery disease to be predictors of readmission. In our study, a medical history of coronary artery disease and diabetes mellitus had a significant effect on readmission risk. However, those with renal impairment did not. However, laboratory values and cardiac investigation data were not analyzed to support these findings.

### 4.4 The Choice of 6‐Month Readmission

While 30‐day readmission rates are often used to as a metric to identify the quality of inpatient healthcare delivery, we evaluated the prognostic value of KCCQ‐12 in predicting longer term outcomes. We felt that a 6‐month readmission window would be more relevant when evaluating patients in an outpatient setting and understanding the prognostic implications of a lower KCCQ‐12 score at this visit could drive changes in medication escalation, follow up frequency and other interventions in the 6‐month window. The 6‐month readmission window is also less studied than shorter readmission periods (i.e., 30‐day and 3‐month).

### 4.5 Limitations

First, the study was conducted in single center with a predominant male enrollment (73%), therefore affecting generalizability.

Next, there is potential risk of selection bias since patients who were non‐compliant to medications or lost to follow‐up, were excluded. Given the likelihood that this group of patients may experience worse outcomes (readmission), excluding them could have underestimated the true readmission rate and predictive capability of the model.

Thirdly, KCCQ scores were only collected at a single time point and not re‐evaluated during the 6 month period; both overall KCCQ scoring and point change have been found to useful in patient evaluation [21]. Furthermore, our study did not include laboratory test data, other patient metrics (including height, weight, systolic blood pressure) or medication dosages. These are potential confounders, and factors that could be incorporated into the risk prediction model.

Lastly, although all patients included had LVEF ≤ 40% on index admission, LVEF information at enrollment or after GDMT optimization was not included in our analysis; LVEF improvement which could have significantly influenced readmissions rate at the chosen longer 6‐month time point. Patients enrolled were also not assessed for their severity of symptoms on presentation, out of the 180 patients enrolled 152 patients were admitted via the emergency department and deemed to have HF as their primary diagnosis.

### 4.6 Future Directions

Our study adds to the wealth of data of the advocating the utility of KCCQ scoring. Although KCCQ has been shown to be more accurate than the commonly used NYHA score [12], it remains an underutilised tool due to challenges in administration and lack of consensus on score interpretation. Large trials have often established ranges and thresholds for their own population [22] which suggests that a population specific or patient specific threshold and range would be most ideal. Despite these challenges KCCQ scores have consistently been shown to predict outcomes supporting the importance of patient reported health statuses in clinical practice. With the increased use of digital tools to facilitate medical care (such as mobile device applications) PROM such as KCCQ could easily administered and even integrated regular clinical practice.

Future research can also seek to improve on the risk prediction model by incorporating other clinically relevant variables such as biochemical laboratory data and LVEF. This model can then be further validated on larger population sample sizes across various centers, locally and overseas. Lastly, given the chronic, long‐term disease course of HF, it is also important to identify patients at risk of readmission beyond just 6 months. In this vein, longer‐term follow up can be conducted on these patients to perform serial measurements of KCCQ, and changes in scores can be incorporated into the model to predict the risk of longer‐term readmissions (e.g., 1‐, 2‐ or 3‐year).

Our study suggest that in our population patients in the “fair to good” health status quartile are at an increased risk of readmission and if patients score below 75 in the overall KCCQ scores closer follow up and monitoring might help mitigate readmissions [23].

## 5 Conclusion

KCCQ‐12 is effective in predicting 6‐month HF readmissions in a mulit‐ethnic Asian population. Our study suggests those with a KCCQ‐12 score in the “fair to good” range were more likely to be readmitted compared with scores 75 to 100.

## Author Contributions

**Jeanne Ong Shan Ying:** conceptualization, methodology, project administration, and writing – original draft. **Kham Ming Fatt:** conceptualization, methodology, formal analysis, validation, project administration, and writing – original draft. **Jonah Goh Yu An:** methodology, formal analysis, validation, writing – review and editing. **Francis Phng Wei Loong:** methodology, data curation, formal analysis, validation, writing – review and editing. **Chan Po Fun:** conceptualization and writing – review and editing. **Poay Huan Loh:** validation, writing – review and editing. **Christine Wu Xia:** methodology, funding acquisition, investigation, data curation, formal analysis, validation, and writing – review and editing.

## Ethics Statement

The study has received ethical approval from the NHG Domain Specific Review Board (DSRB).

## Conflicts of Interest

The authors declare no conflicts of interest.

## Data Availability

The data sets used and/or analyzed during the current study are available from the corresponding author upon reasonable request.
